# Faithful Families Cooking and Eating Smart and Moving for Health: Evaluation of a Community Driven Intervention

**DOI:** 10.3390/ijerph15091991

**Published:** 2018-09-13

**Authors:** Caitlin Torrence, Sarah F. Griffin, Laura Rolke, Kelli Kenison, AltaMae Marvin

**Affiliations:** 1 Department of Public Health Sciences, Clemson University, Clemson, SC 29634, USA; ctorren@clemson.edu (C.T.); lrolke@clemson.edu (L.R.); 2 Health Services Policy and Management, Arnold School of Public Health, University of South Carolina, Columbia, SC 29208, USA; kenison@mailbox.sc.edu; 3 Clemson University Cooperative Extension, Clemson University, Clemson, SC 29634, USA; amarvin@clemson.edu

**Keywords:** dietary intervention, multilevel intervention, diet & exercise, health outcomes

## Abstract

*Background*: There is an increasing need to adapt and use community interventions to address modifiable behaviors that lead to poor health outcomes, like obesity, diabetes, and heart disease. Poor health outcomes can be tied to community-level factors, such as food deserts and individual behaviors, like sedentary lifestyles, consuming large portion sizes, and eating high-calorie fast food and processed foods. *Methods*: Through a social ecological approach with family, organization and community, the Faithful Families Cooking and Eating Smart and Moving for Health (FFCESMH) intervention was created to address these concerns in a rural South Carolina community. FFCESMH used gatekeepers to identify 18 churches and four apartment complexes in low-income areas; 176 participants completed both pre- and post-survey measures. *Results*: Paired *t*-test measures found statistically significant change in participant perception of food security (0.39, *p*-value *=* 0.005, *d* = 0.22), self-efficacy with physical activity and healthy eating (0.26, *p*-value = 000, *d* = 0.36), and cooking confidence (0.17, *p*-value = 0.01, *d* = 0.19). There was not significant change in cooking behaviors, as assessed through the Cooking Behaviors Scale. *Conclusion*: FFCESMH shows that a social ecological approach can be effective at increasing and improving individual healthy behaviors and addressing community-level factors in low-income rural communities.

## 1. Introduction

Diet and exercise have been identified as modifiable behaviors that can reduce poor health outcomes, including obesity, diabetes, and heart disease [1,2,3,4]. However, the prevalence of these diseases, which are sensitive to behavior change, continue to remain high [5]. Obesity and diabetes are increasing around the world. In the United States, one-third of adults are obese [5,6]. Growing portion sizes for meals consumed outside the home, limited access to healthy food choices, and the availability of high-calorie fast-food and processed foods are some explanations for the increase in poor health outcomes in the United States [1]. Living in a food desert or a community with low-access to food is another risk factor for poor health outcomes [7].

The United States Department of Agriculture (USDA) defines food deserts as “parts of country vapid of fresh fruit, vegetables, and other healthful whole foods” [8]. More specifically, if the closest grocery store is at a distance greater than one mile to at least 500 people or 33% of a census tract’s population, the area is considered a “low-access” community. [8]. Communities located within food deserts and low-access areas tend to be poorer and have lower-education levels [7]. In the United States, it is not uncommon for these areas to have a lower population density and be rural. Rural areas have a greater risk of suffering from this affliction [7]. Though rural residents may live near farms or other agricultural endeavors, they often consume fewer fruits and vegetables than their urban peers [8,9]. In South Carolina, where this study takes place, middle- income neighborhoods have, on average, 25% more supermarkets than low-income communities [10]. As a result, fewer fruits and vegetables are consumed in low-income, rural areas [7]. This is particularly concerning as the importance of fruit and vegetable consumption in preventing heart disease and diabetes is well documented [11,12,13,14,15]. 

Poor health outcomes have often been consistently associated with a sedentary lifestyle [16,17,18,19]. Low-levels of energy expenditure, as characteristic of a sedentary lifestyle, have been linked with obesity, diabetes, high blood pressure, and heart disease [18,20,21]. Compounding the concern, there is evidence suggesting rural residents are generally less active than urban residents. Often rural residents have few safe options for engaging in exercise and physical activity [22]. While poor health outcomes are not specific to rural communities [6,23,24], living in a rural area is associated with poorer health outcomes [22,25] and a greater risk of numerous negative health outcomes, including heart disease and type II diabetes [22,25].

Multilevel approaches addressing health problems have been a recommended health promotion practice for more than twenty years [26]. The social ecological framework provides an appropriate lens for addressing behavior change [27]. Individual behavior change is more likely to occur if health promotion programs and activities address the needs of the individual through a multi-layer context that are culturally appropriate. This context must acknowledge and address the individual characteristics, as well as the influencing characteristics of the family, organization, and community within which behaviors occur [26,27]. This is especially pertinent to rural communities where there is a greater risk of a dynamic interplay between individual behaviors and organizational and community level barriers. For example living in a community with low-access to food or limited physical activity resources directly influences the individual’s ability to engage in healthy behaviors [7,25]. Churches have been found to play an important role in improving health within rural communities. This has been especially evident in African American rural communities where religiosity and church attendance tend to be high [28].

Core components of many multilevel approaches to improving obesity related health outcomes focus on nutrition and exercise. Interventions that increase access to fresh fruits and vegetables combined with nutrition education focused on food preparation and cooking are more successful than interventions focused solely on food access or solely on nutrition education [29,30,31,32,33,34,35,36,37,38]. Similarly, faith-based interventions that address access to safe places for physical activity combined with designated time, skill building, and support to be physically active have been shown to increase physical activity [39,40,41]. Based on the above mentioned education and studies, a holistic approach to address healthy behaviors may be beneficial for rural communities. The current study aims to assess the effectiveness of a multi-level intervention, developed through adapting and combining other previously tested effective programs to create a holistic ecological intervention, in improving eating behaviors and increasing physical activity for a rural community in the south eastern area of the United States.

## 2. Materials and Methods

### 2.1. Intervention Education Curriculum Components

Faithful Families Cooking and Eating Smart (FFCESMH), a family-centered multi-level ecological intervention, was created to improve nutrition and physical habits. FFCESMH includes a nutrition and physical activity education component, a mobile farmers market, and technical assistance and resources to area faith-based organizations to address the organizational barrier to healthy eating and physical activity. FFCESMH education components combine the practice proven program, Faithful Families Eating Smart and Moving More (FFESMM), with an evidence based program, Cooking Matters (CM) [37,38,42,43,44,45]. FFESMM is comprised of nine nutrition and physical activity lessons that are designed to be facilitated by lay leaders from within faith organizations [43,44]. Cooking Matters empowers participants with the skills, knowledge, and confidence to make healthy and affordable meals [37,38,42,45]. Following recommendations from the literature [46,47,48], FFESMM and CM curricula were augmented with messaging reinforcing healthy family relationships, social support, and effective parenting styles [49]. Both curricula were modified to emphasize fresh local fruits and vegetables available during the time of lessons. The six FFCESMH lessons typically lasted 90 min and included nutrition tips, recipes, cooking demonstrations, cooking practice activities, physical activity tips, and a structured time for participants to be physically active. Healthy Family Messages reinforced FFFSMM session content and were communicated verbally, through signage, and via “tip sheets” given to families during FFCESMH program sessions.

### 2.2. Intervention Community and Organizational Change Components

In addition to educational components highlighting the importance of nutrition and exercise, FFCESMH included a mobile farmers market. This innovative feature of the program directly addressed community barriers of access to good quality, healthy foods such as fresh fruits and vegetables. The mobile farmers market operated for six-weeks and functioned in the same manner as a traditional ice-cream truck; however, it was stocked with local produce and equipped to accept multiple forms of payment including cash, credit/debit card, and the Supplemental Nutrition Assistance Program (SNAP). The mobile farmers market was run by a retired local community member and it was supported by community businesses and the county farmers market. FFCESMH also included mini-grants of $1000 to each program site. To qualify for a mini-grant award each site had to develop and submit a plan to address organizational barriers to eating healthy and physical activity. Each plan had to include at least one organizational policy change and one structural change. Policy changes could focus on organizational factors, such as guidelines regarding allowable food at organization sponsored events or incorporating physical activity “breaks” during organization meetings and worships. Structural changes could focus on factors such as updating organization kitchens to improve storage for fresh fruits and vegetables or cooking appliances, and improvements to organizational playgrounds, ball courts, ball fields, or walking tracks.

### 2.3. Study Design and Sample

A large, rural South Carolina county, which was also designated as an area of low-access to food at the time of this study, was identified for the implementation of FFCESMH. Over half of the county was designated as a low-access food area [8]. At the time of the study, this county had an adult obesity rate of forty percent, eight percent higher than the state of South Carolina [50]. Working with community gatekeepers in the selected county, 22 sites were selected for delivering the FFCESMH program. The target sites within the county were churches in low-income areas and low-income apartment complexes. FFCESMH was delivered at 18 churches and four low-income housing developments. Churches were selected based on their location within the county with attention paid to their spread around the county and the extent that they were located in rural areas of the county. Organization recruitment focused on churches that had not participated in previous community healthy eating initiatives. The intervention was open to all church participants and residents in the housing sites. Survey participation was a convenience sample from participating churches and housing sites. All of the adults participating in the program at each site were encouraged to complete surveys; however, it was not a requirement for participating in the program. Participants in the evaluation were either a member of a participating church or residing within a specified low- income housing apartment at the time of the study. While the program was designed for adults, some children were eager to attend the programming and allowed to participate. A pre-test survey was administered prior to the start of the first class within the program series and the post-test survey was administered upon conclusion of the series. The six-week program was delivered to each site over the course of one and a half years. The intervention was provided to 410 individuals, among which 176 individuals consented to participate in the study. This study was approved by the Clemson University Institutional Review Board (approval number 2014001418).

### 2.4. Measures

Completeness of intervention implementation was assessed through delivery checklist and attendance records. Intervention fidelity was assessed through session observations by the program evaluators. Intervention outcomes were assessed through participant pre/post surveys. FFCESMH participants completed surveys that included basic demographic questions and assessed a variety of nutrition and physical activity characteristics. Cooking Matters validated assessments were used to assess three diet and behavior constructs including diet patterns, dietary choices, and psychosocial influencers, such as cooking barriers and confidence. The Cooking Matters assessment includes a total of 49 questions [37].

The validated Cooking Matters scales were used to assess the healthy eating segments of the intervention [37]. All scales were implemented and analyzed per the Cooking Mattters guidelines [37] except for the Cooking Confidence Scale. This scale includes four questions that assess participant confidence in cooking and purchasing habits of health foods. Two additional questions to assess cooking confidence were added. The Cronbach’s alpha of the new scale was 0.87. For a list of the constructs measured by the Cooking Matters evaluation see Cooking Matters Course Impact Evaluation Final Report [37].

The Faithful Families Eating Smart and Moving More Self-Efficacy for Healthy Behaviors Scale (Cronbach’s alpha 0.94) was used to further assess confidence with both food selection and engagement in physical activity. While seven individual items were used to assess family support for healthy lifestyle changes [43,44].

To assess physical activity, the validated Rapid Assessment of Physical Activity (RAPA) was incorporated. Participants were provided an example of light, moderate, and vigorous activity and then asked to assess the frequency that they engage in the activity. The RAPA also has an additional component that assesses participant strength and flexibility. The RAPA was implemented and used, as outlined by the assessment developers [51].

### 2.5. Statistical Analysis

Survey responses were analyzed using STATA version 14. Descriptive statistics were used to assess participant demographics and paired *t*-tests were used to assess differences in pretest and posttest means. Only participants who completed both pre and post assessments were included in the analysis. Statistical significance was set at the 0.01 level and the study was powered at 80 percent. Nominal *p*-values of 0.05 are also reported.

## 3. Results

### 3.1. Intervention Delivery

Each FFCESMH session contained an introduction, two nutrition education sessions, a cooking session, social time for participants to eat what they prepared during the cooking session, and a designed time for physical activity. Session instructors used delivery checklists to report the amount of each session that was completed. These results were high, ranging from 75% for the cooking session to 92% for the introduction. Independent program delivery observations that were conducted by evaluators found that delivery adaptations occurred at each site; however, these adaptations did not cause the program to deviate from the core lesson objectives and session goals, thus maintaining program fidelity. Modifications to planned lessons were predominately made because of time shortages or space limitations. Attendance was taken at each session indicating that over 410 individuals participated in the FFCESMH sessions. Each participating organization committed to developing a health plan for their organization and implementing a minimum of two of their planned organizational policy or procedure changes. Nutrition oriented changes mostly focused on limiting soft drinks or sweet tea and encouraging water, less sugar in iced tea, fewer desserts, and processes ensuring that healthy food options were available at all church or housing site sponsored events. Four churches also facilitated a food delivery program to share farmers market items. Fifty-one families were served for nearly two months due to this outreach. Physical activity oriented changes included offering exercise classes, building fitness trails, holding weekly “praise walks”, and updating ballfields.

### 3.2. Sample Characteristics

There were 176 individuals who agreed to participate in the study and completed a pre- and post-test survey (Table 1). Nearly all of the participants identified as female (88.4%) and over half indicated that they were 60 years of age or older (55.9%). While participant age ranged from under 18 to over 60, the majority (83%, 141) identified as 40 years of age or older. Ninety-five percent of participants reported their race as African American. Four percent identified as white and one percent classified as “other” race. Many participants reported having a high school diploma/GED (55, 33.1%) or some college (52, 31.4%). However, nearly thirty percent (45, 27.1%) report having a college or graduate degree. Conversely, 8.4% (14) individuals reported having less than a high school diploma, indicating that while racially homogeneous, educational attainment was quite diverse among our sample. The household size of participants ranged from living alone to living with four or more individuals. Nearly half of the participants lived with at least two additional people (76, 43.9%). Most participants reported living with one additional person (60, 34.7%). While the sample was mostly comprised of middle-age and older adults, over 32% (54) reported that a minor resided within their household. Participants were asked about their household’s use of food-based public assistance, including the Women, Infants, and Children (WIC) program, SNAP, free or reduced-price school breakfasts, lunches, and dinners, free summer meals, Head Start, or if they frequent a food pantry. Forty-one percent of the sample reported that they received or used at least one of the nutrition programs. Of those receiving a form of food-based public assistance, the majority (56.7%) were receiving only one type; however, 16.5% (29) reported supplementing meals with two or more public assistance food programs.

### 3.3. Participant Healthy Eating & Physical Activity Outcomes

#### 3.3.1. Participant Dietary Patterns & Dietary Choices

Participants who completed the six-week program on average increased the frequency that low-fat dairy options were consumed (Table 2). Thirty-six percent of participants at baseline reported “often” or “always” eating low-fat options, while forty percent reported “often” or “always” at the completion of the program. The mean score increased from 3.0 (*sd* = 1.4) on the pretest to 3.3 (*sd* = 1.1) on the posttest. The difference between the two means is statistically significant at the 0.01 level (*t* = −3.16, *df* = 163, *d* = 0.25). The frequency that the participants purchased low-fat meat products also significantly increased (*p*-value = 0.008, *t* = −2.69, *df* = 164, *d* = 0.21). The mean score increased from 3.7 (*sd* = 1.0) at baseline to 4.0 (*sd* = 1.1) at posttest. At baseline, 11% of participants reported that they never or rarely purchase low-fat meats. Upon program completion, only 7% reported never or rarely making these types of purchases. When eating out, the participants reported that they made more frequent attempts to order healthy foods including fruits, vegetables, whole grains, lean meats, low-fat dairy products, and water. The mean score increased from 3.3 (*sd* = 1.2) on the pretest to 3.5 (*sd* = 1.2) on the posttest. The difference between the two means is marginally significant at the 0.05 level (*t* = −2.18, *df* = 167, *d* = 0.17).

#### 3.3.2. Participant Food Resource Management

While mean changes for most questions regarding purchasing healthy food indicated that participants more frequently checked prices prior to purchasing food, most of the changes were insignificant (Table 3). However, participants reported a significant decrease in the frequency that they worry about running out of food before being able to afford to purchase more. At baseline, 18.5% reported “often” or “always” worrying. At program conclusion, 14.8% reported experiencing this worry. The mean score decreased from 2.7 (*sd* = 1.6) on the pretest to 2.3 (*sd* = 1.3) on the posttest. The difference between the two means is statistically significant (*p*-value = 0.005, *t* = 2.80, *df* = 168, *d* = 0.22). In addition to food security, participants reported that they more frequently used “nutrition facts” and food labels when purchasing food. The mean score increased from 3.0 (*sd* = 1.5) on the pretest to 3.40 (*sd* = 1.2) on the posttest. The difference between the two means is statistically significant (*p*-value < 0.001, *t* = −3.80, *df* = 165, *d* = 0.30).

#### 3.3.3. Participant Cooking Behaviors and Confidence

There were not significant changes in the Cooking Behaviors Scale scores; yet, participants were more likely to disagree with the scale items upon program completion (Table 4). Disagreement with the scale items, indicating more positive cooking behaviors, were relatively high (75%) at the baseline. Conversely, the Cooking Confidence Scale resulted in significant changes between the pretest and posttest. The mean score increased from 4.2 (*sd* = 0.90) on the pretest to 4.4 (*sd* = 0.90) on the posttest. The difference between the two means is statistically significant (*p*-value = 0.01, *t* = −2.46, *df* = 170, *d* = 0.19). While cooking confidence was relatively high at baseline (25% reporting “very confident”), participants were more likely to report being very confident (38%) at follow-up. Notably, participants were significantly more confident that they could help their family eat healthier. The mean score increased from 4.3 (*sd* = 1.1) on the pretest to 4.5 (*sd* = 0.91) on the posttest. The difference between the two means is statistically significant at the 0.01 level (*t* = −2.70, *df* = 168, *d* = 0.21).

Participant self-efficacy and confidence associated with increased physical activity and healthy eating habits, as assessed by the self-efficacy scale, also indicated significant improvement (Table 5). The mean score increased from 3.3 (*sd =* 0.74) on the pretest to 3.6 (*sd =* 0.73) on the posttest. The difference between the means is statistically significant (*p*-value *=* 0.000, *t* = −4.39, *df =* 151, *d =* 0.36). Each individual item within this scale was highly significant indicating that on average participants feel more confident in planning and preparing healthy foods and promoting physical activity within the family. While all the items that focused on family support and healthy family initiatives resulted in positive improvements, only one item resulted in marginally significant change. After completing the program, families were more likely to report that they plan how to make healthy meals. The mean score increased from 3.0 (*sd* = 1.2) on the pretest to 3.3 (*sd* = 1.2) on the posttest. The difference between the means is marginally significant at the 0.05 level (*t* = −2.15, *df* = 140, *d* = 0.18).

#### 3.3.4. Participant Physical Activity

Participants reported significant improvements in physical activity and exercise frequency and intensity (Table 6). At baseline, nearly half of the participants assessed with the RAPA were identified as receiving less than the recommended amount and intensity of exercise. For example, 26 percent of participants reported “doing some light physical activity every week”, which is classified as regular underactive. Upon program completion, only 37% of participants were classified as not engaging in enough physical activity. Further, just 6% of participants reported only “doing some light physical activity every week”. The mean reported change in physical activity frequency and intensity increased from 4.8 (*sd* = 1.9) at pretest to 5.3 (*sd* = 1.7) at posttest. The difference between the two means is statistically significant at the 0.01 level (*t* = −2.92, *df* = 156, *d* = 0.23). Participant strength and flexibility scores also improved. The mean score increased from 1.0 (*sd* = 1.2) at pretest to 1.8 (*sd* = 1.8) at posttest. The difference between the two means is statistically significant at the 0.01 level (*t* = −6.05, *df* = 175, *d* = 0.46).

## 4. Discussion

High rates of obesity where the study took place and in other areas of the world illustrate the need for effective community-based health education and promotion. Our evaluation findings are similar to findings from other community-based healthy eating and physical activity program evaluations [29,30,31,32,33,35,36,37,52,53]. Building on previous research, this program took a novel approach to address a key barrier to healthy meal preparation in many communities with low-access to food. In these communities, while nutrition and promotion classes can be effective at increasing healthy behaviors [10,54]; access to healthy food must also be addressed [7,9,10]. Incorporating a mobile farmers market into FFCESMH helped to address this critical barrier for achieving healthy food-related behaviors. By building access to healthy food into the program, participants in this FFCESMH were enabled to apply classroom techniques within their home.

Access is a defining feature of food deserts and low-access areas [7,54]. Especially important to note about access is that it has the great potential to cause a domino effect on resource strain. For instance, as is often the case in rural communities where a lack of cost-effective public transportation is common, individuals must drive a distance to access groceries. This requires access to a car and the longer drive requires gas money that is often more costly than public transportation [10,55]. The expenditures used to access food among lower-income rural individuals may reduce the amount of money that can be spent on food. The mobile farmers market component of this program brought healthy food to local communities, effectively stimulating the implementation of program education. It also likely influenced how monetary resources were utilized and assisted with family food budgeting. A significant finding of this study was that participants were far less likely to worry about running out of food before being able to afford more. Having food brought to the community that can be purchased with SNAP benefits is a community level approach that addresses a fundamental barrier to access and reduces the domino effect brought on by limited resources at the individual, intrapersonal, and community level.

Dissemination and implementation science calls for a focus on assessing the effectiveness of proven effective programs as they are adapted and implemented by lay people in real-world conditions and with different populations [56,57,58]. This study contributes to our understanding of effects when programs are adapted. By combining complementary programs that provide information on how to select healthy foods, instruction on cooking, and establishing an opportunity to practice behaviors, participants experienced significant increases in knowledge and confidence with food preparation. This individual level approach results in participants who are more confident in their ability to prepare healthy meals. While confidence in food preparation did not translate into significant changes in behaviors, the trend was positive and similar to findings from other evaluations of Cooking Matters and FFESMM [42,43,45]. Further, while the education component of this program was only six to nine-weeks and the farmers market was only five weeks, the program that was built on a foundation that the community has sufficiently invested for many years. This program expanded a previous community and state initiative termed Eat Smart Move More (ESMM), which focused on improving health outcomes such as reductions in rates of diabetes and obesity [10]. FFCESMH was implemented in a community heavily invested in the ESMM initiative. Behaviors are often more difficult to alter, and short-term programs are less likely to result in significant behavior change [59]. However, the fact that many of the constructs measured were higher than expected at baseline is likely to be the result of previous community endeavors. For instance, over 75% of participants had positive cooking behaviors at baseline, including disagreement with statements, such as “Cooking takes too much time” or “It is too much work to cook”. Further, the average baseline score for cooking confidence behaviors ranged from 4.1 to 4.3 indicating that participants were “very” confident with their ability to cook. This is higher than baseline scores reported in other Cooking Matters program evaluations [45].

Physical activity, a core component of FFCESMH, was readily incorporated into each level of the social ecological framework. Focusing on the family as well as the individual for many of the physical activity components of the program helped address the influence of social support on motivation. FFCESMH participants reported improvements in physical activity similarly to the FFESMM program [43] and to other faith-based programs that are specifically focused on physical activity [39]. Like many other education programs, self-efficacy for individual factors, such as eating better, resulted in significant changes; this program also resulted in significant changes in confidence of participants to engage their family members and promote healthy behaviors for their loved ones and community [60]. At the organizational level, the program sites developed policies to encourage and support physical activity. Further, it is possible that the previous community endeavors focused on healthy eating primed individuals and the community to accept the physical activity initiative.

While the findings provide valuable insight, there are several limitations. The sample size is small. It is a convenience sample from within the participating organizations and does not include all who were exposed to the intervention. It could be that those who were willing to participate in both the pre and post program survey were different in terms of their level of intervention participation or outcomes when compared to others who did not want to participate in the survey. This project also did not include a control or comparison group. Therefore, we are very careful not to make statements of causation, only statements of difference from pre-intervention to post intervention.

## 5. Conclusions

A social ecological approach to program planning and implementation may be effective at increasing and improving healthy behaviors. Underpinning programs with an understanding of the interplaying factors at various levels will help to tailor programming to the specific needs of the target individuals and the larger community within which they reside. By addressing access to healthy foods as a key component of a healthy eating program, low income rural participants reported less worry about running out of food before being able to afford more. As it has been acknowledged, communities with poor food literacy often need more than education to improve eating behaviors and access to healthy food is a vital component. Bringing healthy, seasonally appropriate food to low-income rural communities will support education programs. Further, communities that have successfully implemented healthy behavior programs may be well poised to build on these programs to include additional healthy behaviors, such as exercise and physical activity. A lengthier follow-up period to this study would help better assess the permanence of the changes. Future studies and programs should explore the unique strengths and weaknesses of the mobile farmer’s market while using the social ecological model to ground the analysis.

## Figures and Tables

**Table 1:** Participant Demographics (n = 176).

**Gender**	**n (%)**
Male	20 (11.6%)
Female	153 (88.4%)
**Age**	
Under 18	6 (3.5%)
18–29	13 (7.7%)
30–39	10 (5.9%)
40–49	18 (10.6%)
50–59	28 (16.5%)
60 and over	95 (55.9%)
**Race**	
White	7 (4%)
Black	164 (95%)
Other	2 (1%)
**Ethnicity**	
Hispanic	1 (0.6%)
**Education**	
Less than high school	14 (8.4%)
High school degree/GED	55 (33.1%)
Some college/2-year degree	52 (31.4%)
College degree (4 year)	20 (12.1%)
Graduate degree	25 (15.0%)
**Household size**	
Live alone	37 (21.4%)
Live with 1 person	60 (34.7%)
Live with 2 persons	31 (17.9%)
Live with 3 persons	21 (12.1%)
Live with 4 or more persons	24 (13.9%)
Minor in household	54 (32%)
**Public assistance**	67 (41.9%)
Women, Children, and Infant (WIC)	11 (6.3%)
Supplemental Nutrition Assistance Program (SNAP)	41 (23.3%)
Free or reduced-price school breakfast	22 (12.5%)
Free or reduced-price school lunch	25 (14.2%)
Free or reduced-price school supper	3 (1.7%)
Free summer meals	12 (6.8%)
Head Start	5 (2.8%)
Food pantry	12 (6.8%)
**Number of different types of public assistance**	
One	38 (56.7%)
Two or more	29 (43.3%)

**Table 2:** Dietary Patterns & Choices Mean Change.

Survey Items or Scales	Mean (SD)	
	Baseline	6-Week (Post)
**Dietary Patterns Scale**	2.7 (0.5)	2.7 (0.4)
How often do you typically eat fruit like apples, bananas, melon, or other fruit?	3.3 (1.1)	3.4 (1.0)
How often do you typically eat green salad?	2.6 (0.90)	2.8 (0.90)
How often do you typically eat French fries or other fried potatoes, like home fries, hash browns, or tater tots?	2.1 (0.77)	2.0 (0.76)
How often do you typically eat any other kind of potatoes that aren’t fried?	2.1 (0.80)	2.0 (0.86)
How often do you typically eat refried beans, baked beans, pinto beans, black beans, or other cooked beans?	2.0 (0.90)	2.1 (0.87)
How often do you typically eat other non-fried vegetables like carrots, broccoli, green beans, or other vegetables?	2.9 (0.94)	3.0 (0.91)
How many times a week do you typically eat a meal from a fast-food or sit- down restaurant? (consider breakfast, lunch and dinner.)	2.3 (0.84)	2.1 (0.80)
How often do you typically drink 100% fruit juices like orange juice, apple juice or grape juice?	2.8 (1.1)	3.0 (1.1)
How often do you typically drink a can, bottle or glass of regular soda or pop, sports drink, or energy drink?	2.3 (1.2)	2.3 (1.2)
How often do you typically drink a bottle or glass of water?	4.5 (0.89)	4.5 (.84)
**Dietary Choices**		
When you have milk, how often do you choose low-fat milk (skim or 1%)?	3.0 (1.6)	2.9 (1.5)
When you eat dairy products like yogurt, cheese, cottage cheese, sour cream, etc., how often do you choose low fat or fat-free options?	3.0 (1.4)	3.3 (1.1) **
When you eat grain products like bread, pasta, rice, etc., how often do you choose whole grain products?	3.3 (1.2)	3.5 (1.2)
How often do you choose low-sodium options when you buy easy-to-prepare, pre-packaged foods like canned soups or vegetables, pre-packaged rice, frozen meals, etc.?	3.1 (1.3)	3.3 (1.2) *
When you buy meat or protein foods, how often do you choose lean meat or low-fat proteins like poultry or seafood (not fried), 90% or above lean ground beef, or beans?	3.7 (1.0)	4.0 (1.1) **
When you eat at fast-food or sit-down restaurants, how often do you choose healthy foods? (Healthy foods include fruits, vegetables, whole grains, lean meats, low-fat or fat-free dairy, and water.)	3.3 (1.2)	3.5 (1.2) *

* *p*-value ≤ 0.05, ** *p*-value ≤ 0.01, *** *p*-value ≤ 0.001.

**Table 3:** Food Management Mean Changes.

Healthy Food Preparation	Baseline Mean (SD)	6-Week Post Mean (SD)
How often do you compare prices before you buy food?	4.0 (1.3)	4.1 (1.1)
How often do you plan meals ahead of time?	3.4 (1.3)	3.2 (1.1)
How often do you use a grocery list when you go grocery shopping?	3.5 (1.4)	3.4 (1.3)
How often do you worry that your food might run out before you get money to buy more?	2.7 (1.6)	2.3 (1.3) **
How often do you use the “nutrition facts” on food labels?	3.0 (1.5)	3.4 (1.2) **
How often do you eat breakfast within two hours of waking up?	3.3 (1.4)	3.4 (1.2)
How often do you eat food items from each food group every day?	3.5 (1.2)	3.7 (1.0)
How often do you make homemade meals “from scratch” using mainly basic who ingredients like vegetables, raw meats, rice, etc.?	3.7 (1.3)	3.5 (1.3)
How often do you adjust melas to include specific ingredients that are more “budget-friendly”, like on sale or in your refrigerator or pantry?	3.5 (1.3)	3.5 (1.2)
How often do you adjust meals to be more healthy, like adding vegetables to a recipe, using whole grain ingredients, or baking instead of frying?	3.6 (1.2)	3.7 (1.1)

* *p*-value ≤ 0.05, ** *p*-value ≤ 0.01, *** *p*-value ≤ 0.001.

**Table 4:** Cooking Behaviors and Confidence Mean Changes.

Cooking Behaviors and Confidence	Baseline Mean (SD)	6-Week Post Mean (SD)
**Cooking Behaviors Scale (Scale Items Below)**	2.1 (0.94)	2.0 (0.94)
Cooking takes too much time.	2.2 (1.0)	2.1 (1.0)
Cooking is frustrating.	2.0 (0.98)	1.9 (0.86)
It is too much work to cook.	2.1 (1.0)	2.0 (1.0)
**Cooking Confidence Scale (Scale Items Below)**	4.2 (0.90)	4.4 (0.90) **
How confident are you that you can use the same healthy ingredient in more than one meal?	4.1 (1.2)	4.3 (1.1) *
How confident are you that you can choose the best-priced form of fruits and vegetables (fresh, frozen, or canned)?	4.1 (1.1)	4.3 (1.1)
How confident are you that you can use basic cooking skills, like cutting fruits and vegetables, measuring out ingredients, or following a recipe?	4.3 (1.2)	4.4 (1.1)
How confident are you that you can buy healthy foods for your family on a budget?	4.1 (1.2)	4.4 (1.0) *
How confident are you that you can cook healthy foods for your family on a budget?	4.2 (1.1)	4.3 (1.2)
How confident are you that you can help your family eat more healthy?	4.3 (1.1)	4.5 (0.91) **

* *p*-value ≤ 0.05, ** *p*-value ≤ 0.01, *** *p*-value ≤ 0.001.

**Table 5:** Self Efficacy and Family Support Mean Changes.

Self-Efficacy and Family Support for Health Behaviors	Baseline Mean (SD)	6-Week Post Mean (SD)
**Self-Efficacy for Healthy Behaviors Scale (Scale Items Below)**	3.3 (0.74)	3.6 (0.73) ***
How confident are you in preparing fresh vegetables as part of a meal?	3.8 (0.90)	4.0 (0.80) **
How confident are you in preparing fruits as part of a meal?	3.6 (1.1)	3.9 (0.90) **
How confident are you in using herbs and spices as part of a meal?	3.5 (1.0)	3.8 (1.0) *
How confident are you that you can find ways to exercise or be physically active?	3.7 (0.92)	3.9 (0.84) *
How confident are you that you can reach your exercise or be physically active goals?	3.5 (0.95)	3.8 (0.87) **
How confident are you that you can overcome things that get in the way of exercise or physical activity?	3.4 (0.97)	3.6 (1.0) **
How confident are you that you can get others to exercise with you?	2.9 (1.1)	3.2 (1.1) **
How confident are you that you can find ways to be active with your family?	3.1 (1.1)	3.4 (1.1) **
How confident are you that you can be active with your children?	3.0 (1.3)	3.3 (1.3) **
How confident are you that you can be active with others in your community?	2.9 (1.1)	3.3 (1.1) **
How confident are you that you can be active with others in your church?	3.3 (1.1)	3.5 (1.0) **
**Family Support**		
My family encourages me to make healthy meals.	3.4 (1.1)	3.5 (1.1)
My family helps me make healthy meals.	3.2 (1.1)	3.2 (1.1)
My family and I plan how to make healthy meals.	3.0 (1.2)	3.3 (1.2) *
Our family regularly eats fast food.	2.5 (1.0)	2.6 (.90)
My child (or children) frequently drinks soda or other sweet drinks.	1.5 (1.5)	1.6 (1.4)
My child rarely drinks low-fat milk.	1.7 (1.7)	1.8 (1.7)
Our family does not play games outside, ride bikes, or walk together very often.	2.6 (1.2)	2.4 (1.3)

* *p*-value ≤ 0.05, ** *p*-value ≤ 0.01, *** *p*-value ≤ 0.001.

**Table 6:** Physical Activity Mean Changes.

Rapid Assessment of Physical Activity (RAPA)	Baseline Mean (SD)	6-Week Post Mean (SD)
General Assessment	4.8 (1.9)	5.3 (1.7) **
Rapid Assessment of Physical Activity (2): strength & flexibility	1.0 (1.2)	1.8 (1.8) ***

* *p*-value ≤ 0.05, ** *p*-value ≤ 0.01, *** *p*-value ≤ 0.001.
